# Validation of computational models to characterize cumulative intake curves from video-coded meals

**DOI:** 10.3389/fnut.2023.1088053

**Published:** 2023-07-31

**Authors:** Alaina L. Pearce, Timothy R. Brick

**Affiliations:** ^1^Social Science Research Institute, Pennsylvania State University, University Park, PA, United States; ^2^Department of Nutritional Sciences, Pennsylvania State University, University Park, PA, United States; ^3^Department of Human Development and Family Studies, Pennsylvania State University, University Park, PA, United States; ^4^Institute for Computational and Data Sciences, Pennsylvania State University, University Park, PA, United States

**Keywords:** meal microstructure, cumulative intake curves, eating rate, satiation, mathematical model

## Abstract

**Introduction:**

Observational coding of eating behaviors (e.g., bites, eating rate) captures behavioral characteristics but is limited in its ability to capture dynamic patterns (e.g., temporal changes) across a meal. While the Universal Eating Monitor captures dynamic patterns of eating through cumulative intake curves, it is not commonly used in children due to strict behavioral protocols. Therefore, the objective of this study was to test the ability of computational models to characterize cumulative intake curves from video-coded meals without the use of continuous meal weight measurement.

**Methods:**

Cumulative intake curves were estimated using Kisslieff’s Quadratic model and Thomas’s logistic ordinary differential equation (LODE) model. To test if cumulative intake curves could be characterized from video-coded meals, three different types of data were simulated: (1) Constant Bite: simplified cumulative intake data; (2) Variable Bite: continuously measured meal weight data; and (3) Bite Measurement Error: video-coded meals that require the use of average bite size rather than measured bite size.

**Results:**

Performance did not differ by condition, which was assessed by examining model parameter recovery, goodness of fit, and prediction error. Therefore, the additional error incurred by using average bite size as one would with video-coded meals did not impact the ability to accurately estimate cumulative intake curves. While the Quadratic and LODE models were comparable in their ability to characterize cumulative intake curves, the LODE model parameters were more distinct than the Quadradic model. Greater distinctness suggests the LODE model may be more sensitive to individual differences in cumulative intake curves.

**Discussion:**

Characterizing cumulative intake curves from video-coded meals expands our ability to capture dynamic patterns of eating behaviors in populations that are less amenable to strict protocols such as children and individuals with disordered eating. This will improve our ability to identify patterns of eating behavior associated with overconsumption and provide new opportunities for treatment.

## 1. Introduction

Observational coding of meal eating behaviors (e.g., bites, eating rate) provides the ability to assess complex patterns of behavior within a meal. For example, pediatric obesity has been associated with an “obesogenic” style of eating characterized by larger bites, faster eating and bite rates, and shorter meal durations (1, 2). These behaviors, termed meal microstructure, were originally assessed in observationally-coded animal studies on behavioral and physiological control of food intake (3–5). However, observational coding is limited in its ability to capture dynamic patterns (i.e., temporal changes) within a meal as it relies on averaged eating or bite rates. In contrast, the Universal Eating Monitor developed by Dr. Harry Kissileff assessed dynamic patterns of meal microstructure by continuously measuring food weight over the course of a meal (6). Similarly, a drinkometer was recently developed that provides continuous weight measurement of liquid meals and has been used to investigate differences in meal microstructure after surgery-induced weight loss (7, 8). When graphed over time, continuous weight measurements create a cumulative intake curve (6), which is another quantification meal microstructure that differs in adults with obesity and disordered eating (4, 9–14). Both the Universal Eating Monitor and the drinkometer provide precise measurement of meal microstructure, however, the underlying technology of continuous weight measurement presents unique challenges for a pediatric population.

The use of continuous food weight measurement is uncommon in children (2) due, in part, to two key challenges. First, behavioral protocols required to continuously measure food weight during eating may be difficult for children to follow (e.g., not touching the plate) and restrict typical eating behaviors (e.g., playing with food). Second, it is uncommon to use multi-item meals with Universal Eating Monitors (14, 15) and the “drinkometer” requires the use of liquid meals (8). This limits the utility of these protocols in studies with children, where the standard is to serve multi-item meals (2). Some studies have utilized multiple scales or added observational coding to continuous weight measurement when using multi-item meals (16, 17), however, this substantially increases researcher burden. A possible solution is to use observational coding to characterize cumulative intake curves from bite timing and average bite size. Observational coding is common in studies of eating behavior (2). In the case of children, this approach commonly relies on recording the precise timing of each bite of food in the meal videos (2). Although the size/weight of each bite can only be approximated from video, it remains unclear if average bite sizes can be used to accurately characterize cumulative intake curves.

**FIGURE 1:**
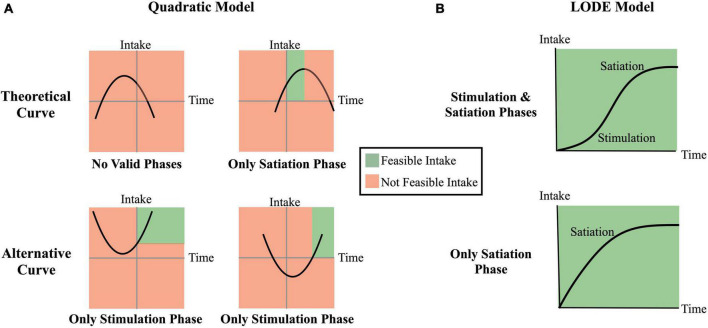
Theoretical comparison of the quadratic model and the logistic ordinary differential equation (LODE) model. Regions shaded in green reflect theoretically feasible intake while regions shaded in red reflect theoretical infeasible (e.g., negative) intake. **(A)** Examples of Quadratic model cumulative intake curves; **(B)** Examples of LODE model cumulative intake curves.

This study will test the ability to characterize cumulative intake curves from average bite sizes using the two existing computational models: (1) a quadratic model (Eq. 1) proposed by Kissileff et al. (18); and (2) a logistic ordinary differential equation (LODE; Eq. 2) proposed by Thomas et al. (19). A key distinction between these models is that while quadratic functions may predict non-feasible cumulative intake patterns (e.g., reductions in intake at the end of the meal; see Figure 1), the first-principles approach used to develop the LODE model requires estimated curves to closely reflect biologically plausible patterns of intake (19). This study aims to improve the applicability of cumulative intake curves by: (1) comparing the use of averaged and measured bites; (2) validating the LODE model; and (3) comparing performance of Quadratic and LODE models. The ability to use average bite sizes would expand the tools available to assess cumulative intake curves in different populations and meal scenarios (e.g., non-laboratory, multi-item).

## 2. Materials and methods

### 2.1. Cumulative intake curve models

#### 2.1.1. Quadratic model

Kissilef et al. (18) identified the quadratic model as the best fitting model for cumulative intake curves (Eq 1).


(1) $$\begin{array}{c} E\,\left(t\right)=a^{2}+b+c \end{array}$$

E(t) is total gram intake at time *t* during the meal. The linear coefficient (*b*) reflects the eating rate, the quadratic coefficient (*a*^2^) reflects the change in eating rate across an eating episode, and the intercept (*c*) is a non-interpreted term for the fit of the line.

#### 2.1.2. LODE model

Thomas et al. (19) used a first-principles approach to propose a new model for characterizing cumulative intake curves that met three key theoretical assumptions (19): (1) eating rate depends on total weight consumed at each point throughout the eating episode; (2) the initial phase of eating includes a short stimulatory period where eating rate is proportional to amount consumed; and (3) a second phase later in the meal is characterized by reduced eating rate due to satiation. The LODE model (Eq. 2) was derived from equations in Thomas et al. (19) (see Supplementary materials) and can take a similar form to exponential decay with $k=\frac{E_{m\,a\,x}\,r+\theta }{E_{m\,a\,x}}$.


(2) $$\begin{array}{c} E\,\left(t\right)=\frac{e^{k\,t}-1}{\frac{e^{k\,t}}{E_{m\,a\,x}}+\frac{r}{\theta }} \end{array}$$

E(t) is total gram intake at time *t* during the meal, *E*_*max*_ is equal to total gram intake during the eating episode, θ is a non-zero initial rate of eating, which we term the *initial state* or *state*, and *r*, which we term *doubling rate* or *rate*, reflects eating duration as $\frac{1}{r}$ approximates the time it takes to double food intake. Parameters *r* and θ are independent. While the s-shape curve defined by the LODE model allows it to capture both stimulatory and satiating periods of eating, only the “top” or “satiating” portion is usually predicted when modeling intake (Figure 1B) (14, 19).

#### 2.1.3. Theoretical comparison (Figure 1)

There are three primary distinctions between the Quadratic and LODE models: (1) the LODE model can simultaneously capture the stimulation and the satiating phases of eating while the Quadratic model can only capture one phases at a time; (2) LODE model predicted intake asymptotes at total grams consumed while the Quadratic model inverted-U shape predicts eventual decline in total intake; and (3) the LODE model parameters are independent, allowing them to capture unique information about the cumulative intake curves.

### 2.2. Simulation process and model validation

**FIGURE 2:**
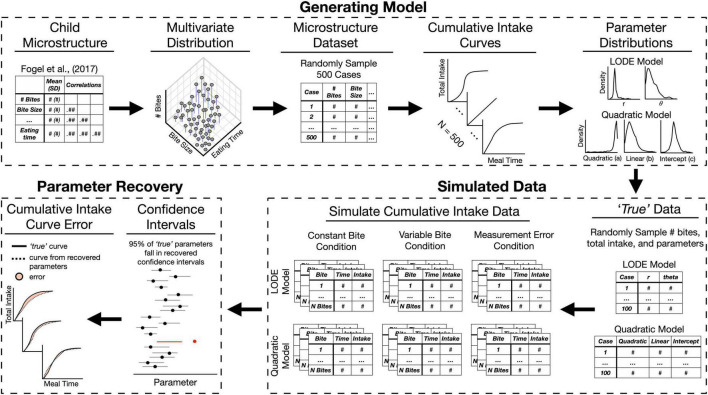
Overview of the simulation process and model validation. The Generating Model section depicts steps described in section “2.2.1. Generating model.” The Simulation section depicts steps described in “2.2.2. Simulated data” (for detailed depiction of simulation conditions see Figure 3). The Parameter Recovery section depicts the two of the approaches used for validating parameter recovery described in section “2.2.3. Parameter recovery.” LODE, logistic ordinary differential equation.

As with any new method, the LODE model needs to be validated to ensure the model estimates can reasonably be interpreted as reflecting the participants’ *true* values. Since we cannot rely on known tests (e.g., *t*-tests), we must rely on confidence intervals to determine if estimates differ from each other. To validate a model, we need to show that a model estimate does not differ from the *true* value by showing it falls within the estimated confidence interval. Therefore, remainder of the section “2. Materials and methods” is focused on how we generated and simulated a reasonable set of data in order to estimate confidence intervals to evaluate Quadratic and LODE model performance (Figure 2). In section “2.2.1. Generating model,” we discuss the process used to establish a reasonable set of cumulative intake patterns for children and identify the associated Quadratic and LODE parameter values. In section “2.2.2. Simulated data,” we outline how we simulate data needed for this study. Finally, in section “2.2.3. Parameter recovery,” we discuss how simulated data were used to estimate parameter confidence intervals. Analyses are publicly available (osf.io/xfk5w/) and were completed in R (20) using the bitemodelr package (21).

#### 2.2.1. Generating model

In order to be reasonably sure the intake data generated would reflect child eating behaviors, we first generated distributions of microstructure behaviors based on characteristics (e.g., means) of children’s behaviors reported in Fogel et al. (1). This study was chosen as a reference because it reported a seven different eating behaviors while most studies in children only report only one or two behaviors (2). Using this set of child microstructure behaviors, we generated cumulative intake curves to establish distributions of Quadratic and LODE model parameters that reasonably characterize child eating behavior (see Figure 2).

We first sought to create a set of distributions from which to generate data by referencing central tendencies and variability reported in Fogel et al. (1). We began with 500 cases (see Supplementary materials). Cumulative intake curves were generated using total number of bites, average bite size, and meal duration. Each bite was set to the average bite size and bite timings were generated by randomly sampling points (*n* = number of bites) from a logistic distribution truncated at zero (22) to approximate the theoretical shape of cumulative intake curves. Sampled points were the scaled to reflect meal timing: $m\,e\,a\,l\,d\,u\,r\,a\,t\,i\,o\,n\,\left(\frac{s\,a\,m\,p\,l\,e\,d\,v\,a\,l\,u\,e}{\text{max}\,\left(s\,a\,m\,p\,l\,e\,d\,v\,a\,l\,u\,e\right)}\right)$. Lastly, bite timings were jittered to allow for variability in cumulative intake curves around the referenced distribution. All bite timing data were validated to ensure: (1) bite times were less than or equal to meal duration, (2) bite timings were positive, and (3) meal time increased successively at each bite (i.e., *t* < *t*+1). This approach allowed eating rates to vary across the meal and resulted in variable cumulative intake curve shapes. After generating cumulative intake curves for each of the 500 cases in the microstructure dataset, parameter estimates were fit for both the Quadratic and LODE models using an iterative process until parameter fit was stable and parameters predicted feasible intake patterns (see Supplementary materials). This resulted in distributions of Quadratic and LODE model parameters that captured reasonable patterns of child meal microstructure and cumulative intake.

#### 2.2.2. Simulated data

**FIGURE 3:**
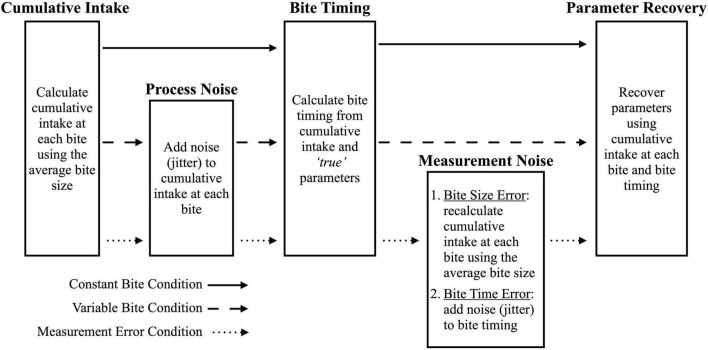
Process used to simulate data conditions. Solid arrows indicate the steps for the constant bite condition, dashed arrows indicate the steps for the variable bite condition, and the dotted arrows indicate the steps for the Measurement Error condition.

To simulate data, we randomly selected model parameters from our distribution of feasible values (see “2.2.1. Generating model”). For each model, 100 samples were randomly drawn from the generated multivariate normal distribution because it is a typical sample size for eating behavior studies in children (2). The randomly selected parameter values were considered to be the *true* parameters for all analyses (Figure 2). Using these *true* parameters, cumulative intake data were simulated according to three different conditions (see Figure 3): (1) Constant Bite: simplified cumulative intake data with constant bite size; (2) Variable Bite: simulated continuously measured intake with variable bite size; and (3) Bite Measurement Error: simulated video coded data with variable bite size and measurement noise added to both bite size and bite timing. These conditions allowed us to determine if measurement error due to using average bite size impacts the ability to characterize cumulative intake curves.

##### 2.2.2.1. Constant bite condition

This condition simulated simplified cumulative intake data which do not reflect human behavior as people do not have constant bite sizes. For each model, 100 samples were randomly drawn from the multivariate normal distribution for number of bites, total intake in grams, and model-specific parameters (e.g., *E*_*max*_, θ, and *r* for the LODE model; see “2.2.1. Generating model”). This resulted in a test dataset for each model which were treated as the *true* parameters for the cumulative intake curves. These datasets were used to simulate cumulative intake data for each model by calculating two variables: (1) cumulative intake at each bite using average bite size and number of bites; and (2) bite timings using the *true* parameters and cumulative intake at each bite. Since the bite timing was calculated directly from the *true* parameters and cumulative intake, there was no measurement error in this condition.

##### 2.2.2.2. Variable bite condition

This condition simulated an idealized case of continuous weight measurement with bites that varied in size. After following the Constant Bite simulation procedure, process noise (i.e., jitter) was added to cumulative intake at each bite so that bite sizes would vary, which better approximates of human eating behavior (Figure 3). There was no measurement error because bite timing was calculated from cumulative intake at each bite.

##### 2.2.2.3. Bite measurement error condition

This condition simulates video coded bite data which require the use of average bite size and has less precise bite timings. After following the Variable Bite simulation procedure, measurement noise was added in two ways *after* bite timings were calculated: (1) cumulative intake at each bite was recalculated using average bite size, reflecting measurement error incurred when using average bite size; and (2) bite timings were jittered to reflect additional error from manually coding bite timing. Since error was added *after* calculation bite timings, both cumulative intake and bite timing have measurement error.

#### 2.2.3. Parameter recovery

Quadratic and LODE model performance was assessed by examining how closely the recovered cumulative intake curves matched the *true* cumulative intake curves. In sections “2.2.3.1. “Confidence intervals” and “2.2.3.2. Distinguishability,” we discuss how confidence intervals were used to test the accuracy of recovered parameter estimates and whether estimates could be used to distinguish different cases. In sections “2.2.3.3. Goodness of Fit Index” and “2.2.3.4. Cumulative intake curve error,” we discuss how we measured error in recovered parameters and cumulative intake curves.

##### 2.2.3.1. Confidence intervals

To determine if the parameter estimates reflect the *true* values, we tested if the *true* parameter fell within the 95% confidence bounds of the recovered estimate. To do so, we first recovered parameter estimates for the Quadratic and LODE models and then constructed data-driven likelihood profile confidence intervals (23) by identifying the upper and lower bounds of the interval for each parameter using an iterative process (see Supplementary materials). This approach was chosen because likelihood profile confidence intervals are more robust than standard error-based approaches for arbitrarily complex models (24, 25). Parameter recovery was assessed by the proportion of *true* values that fell within recovered confidence bounds for each parameter, which we would expect to be 95 of the 100 *true* values for a 95% confidence interval.

##### 2.2.3.2. Distinguishability

Confidence intervals can also be used to determine whether estimates are distinct from each other. When a value falls outside an estimate’s confidence interval, it can be interpreted as being distinct from the estimate. Therefore, to index the distinctness of each recovered estimate, we counted the number of times the parameter estimate fell within the confidence intervals of other estimates in the same condition (i.e., Constant Bite, Variable Bite, or Bite Measurement Error). A parameter estimate was considered distinct if it was distinguishable from 85% of the estimates in the simulated condition (i.e., falls within less than 15 other confidence intervals). Low distinguishability indicates the same cumulative intake data could have been used to recover all the different parameter estimates, which reduces power and ability to use parameter estimates as predictors of individual differences or in interactions. In essence, this is an estimate of parameter variability relative to the precision of the parameter.

##### 2.2.3.3. Goodness of Fit Index

Goodness of fit was calculated by scaling the difference between the recovered and *true* parameters by the median of the parameter distribution (see “2.2.1. Generating model”). Scaling by the median allows for comparisons across parameters that differ in magnitude. We used the absolute value since parameters differ in whether they are expected to be positive (e.g., linear coefficient) or negative (e.g., quadratic coefficient). Therefore, goodness of fit was always positive with smaller values indicating better fit.

##### 2.2.3.4. Cumulative intake curve error

To estimate error in recovered cumulative intake curves, the *true* and predicted cumulative intake or timings were compared across bites. Predicted cumulative intake was calculated for each bite using the recovered parameters and the *true* bite timing while predicted bite timing was calculated using the recovered parameters and *true* cumulative intake at each bite. Root mean squared error (RMSE) was calculated to compare *true* cumulative intake or timing for each bite with the values predicted from recovered parameter estimates. RMSE reflects raw error and has the benefit of maintaining units for interpretation. To index predictive value, a pseudo-*R*^2^ was used to determine the proportion of variance in *true* cumulative intake or bite timing explained by the predicted values. Due to approximation error in recovered parameters, non-feasible predicted values were possible (e.g., intake before the meal began, negative intake at the beginning of the meal, bites resulting in negative intake, etc.) and a model was considered non-convergent if more than 10% of bites were non-feasible (see Supplementary materials).

### 2.3. Analyses

Differences between models and model parameters were tested for distinguishability, goodness of fit, and error in the cumulative intake curve. Since distinctness was a binary outcome, Fisher’s Exact Probability Test was used to test the differences the in proportion distinct. Due to skewed distributions, Mann–Whitney U tests were used for goodness-of-fit and error.

## 3. Results

### 3.1. Descriptive model comparisons (Figure 4)

**FIGURE 4:**
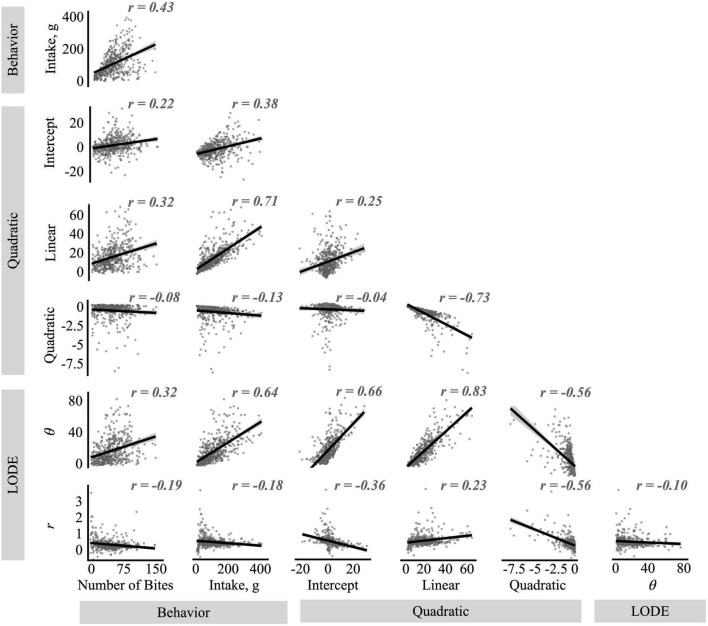
Correlations between the quadratic and logistic ordinary differential equation (LODE) parameters and number of bites and total intake. The *r* values reflect Pearson’s correlation values.

As expected from the theoretical basis of the LODE model, initial state (θ) and doubling rate (*r*) showed very low correlation with each other. In contrast, the quadratic and linear coefficients from the Quadratic model showed a strong negative correlation with each other. While the intercept was not correlated with the quadratic coefficient, it was moderately correlated the linear coefficient. Looking across models, state (θ) was strongly correlated with all Quadratic model parameters while rate (*r*) was moderately correlated with them. Both state (θ) and rate (*r*) were positively correlated with the linear coefficient and negatively correlated with the quadratic coefficient, however, the correlation between rate (*r*) and the quadratic coefficient appeared to be influenced by outlier values (Figure 4). In contrast, while state (θ) was positively correlated with the Quadratic model’s intercept, rate (*r*) was negatively associated it. Similarly, while state (θ) had strong positive correlations with both number of bites and total intake (grams), rate (*r*) was not linearly associated with meal behaviors. The linear coefficient from the Quadratic model was also correlated with number of bites and total intake, while the quadratic coefficient was not correlated with meal behaviors.

### 3.2. Parameter recovery

Due to skewed distributions, the median and 25th and 75th percentiles were used for descriptive statistics (see Supplementary Figures 1–3).

#### 3.2.1. Confidence intervals

**FIGURE 5:**
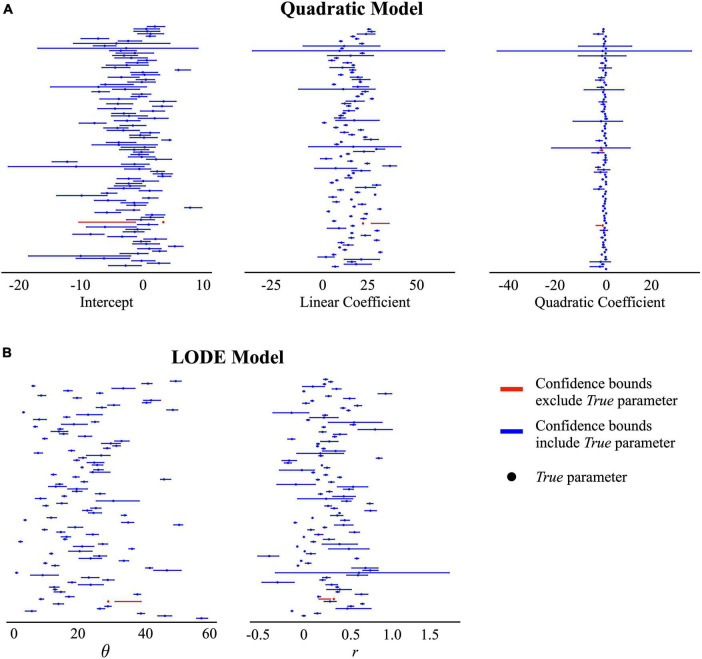
95% confidence intervals for the recovered estimates in the Measurement Error condition. Points reflect the *true* parameter value and lines indicate the recovered 95% confidence interval. The color blue indicates the confidence bounds included the *true* parameter value and the color red indicates the confidence bounds did not include the *true* parameter value. **(A)** Quadratic model estimates: intercept, linear coefficient, and quadratic coefficient. **(B)** Logistic ordinary differential equation (LODE) model estimates: θ and *r.*

Across all conditions, the both models had greater the expected 95% recovery of the *true* parameters by the 95% confidence intervals (Table 1). Recovery that exceeds the expected 95% indicates that the type-1 error rate is slightly less than expected for the recovered 95% confidence intervals (i.e., α < 0.05). The widths of the recovered confidence intervals were small (Figure 5), indicating that even small effects could be distinguished. Ultimately, this indicates that the recovered 95% confidence interval is a slightly more conservative way to test differences between estimates. This shows that both the Quadratic and LODE models are able to recover estimates that reflect *true* parameter values when bite size is measured (i.e., similar to continuous weight measurement) and when average bite size is used (i.e., similar to video coded bites).

**TABLE 1:** Parameter recovery for the quadratic and logistic ordinary differential equation (LODE) models.

Precision of recovered parameters					
	**Quadratic model**			**LODE model**	
	**Quadratic (*a*)**	**Linear (*b*)**	**Intercept (*c*)**	**θ**	***r***
**Distinct estimates, %**					
Constant bite	12%	41%	8%	80%	42%
Variable bite	13%	43%	8%	83%	41%
Bite Measurement Error	12%	46%	11%	84%	41%
**CIs overlapping estimate, med (25th, 75th)**					
Constant bite	34.0 (22.0, 40.0)	19.0 (11.0, 22.0)	42.0 (34.0, 47.0)	10.0 (7.0, 15.0)	17.0 (13.0, 23.0)
Variable bite	34.5 (34.0, 40.3)	18.5 (11.0, 22.0)	41.0 (33.8, 48.0)	11.0 (7.8, 15.0)	16.0 (13.0, 23.0)
Bite Measurement Error	33.0 (21.0, 34.0)	17.5 (10.0, 20.0)	38.0 (25.8, 42.0)	11.0 (6.8, 14.0)	17.0 (12.8, 21.3)
**Goodness of Fit Index, med (25th, 75th)**					
Constant bite	0.11 (0.05, 0.28)	0.04 (0.02, 0.08)	0.53 (0.26, 0.81)	0.21 (0.12, 0.49)	0.07 (0.03, 0.10)
Variable bite	0.11 (0.05, 0.28)	0.04 (0.02, 0.07)	0.45 (0.24, 0.72)	0.20 (0.09, 0.43)	0.07 (0.03, 0.11)
Bite Measurement Error	0.11 (0.04, 0.25)	0.03 (0.01, 0.06)	0.33 (0.18, 0.63)	0.20 (0.09, 0.39)	0.06 (0.03, 0.10)
**Cumulative intake curve error**					
	**Quadratic Model**		**LODE Model**		
	**Timing**	**Intake**	**Timing**	**Intake**	
Constant bite	0.06 (0.04, 0.08)	0.71 (0.71, 0.71)	1.13 (0.76, 1.88)	0.71 (0.71, 0.71)	
Variable bite	0.05 (0.04, 0.07)	0.71 (0.71, 0.71)	1.20 (0.74, 1.88)	0.71 (0.71, 0.71)	
Bite Measurement Error	0.05 (0.03, 0.07)	0.67 (0.60, 0.70)	1.20 (0.75, 1.95)	0.66 (0.60, 0.71)	
**Psuedo-*R*^2^, med (25th, 75th)**					
Constant bite	1.00 (0.999, 1.00)	1.00 (0.999, 1.00)	0.91 (0.88, 0.94)	1.00 (1.00, 1.00)	
Variable bite	1.00 (0.999, 1.00)	1.00 (0.999, 1.00)	0.91 (0.88, 0.94)	1.00 (1.00, 1.00)	
Bite Measurement Error	1.00 (0.999, 1.00)	1.00 (1.00, 1.00)	0.91 (0.88, 0.94)	1.00 (1.00, 1.00)	

CIs, confidence intervals; med, median; RMSE, root mean squared error; 25th: 25th quartile; 75th: 75th quartile.

#### 3.2.2. Distinguishability

Across all simulation conditions, the LODE model parameters were more distinct than the Quadratic model parameters (Table 1). Across conditions, both LODE parameters were more distinct than the Quadratic model’s intercept (*p*’s < 0.001) and quadratic (*p*’s < 0.001) coefficients but only state (θ) was more distinct than the linear coefficient (*p*’s < 0.001). It is also evident in Figure 5 that there is greater overlap of confidence intervals for the individual parameters of the Quadratic compared to the LODE model. Because cumulative intake curves require all model parameters to be defined and characterized, one could argue that a cumulative intake curve would be distinct if *any* of the model parameters were distinct. Although considering all parameters for each model increased the percent of distinct cases, the pattern of results remained the same. Specifically, 46–53% of the cases were distinct for the Quadratic model (Constant Bite = 46%, Variable Bite = 49%, Bite Measurement Error = 53%) while 89–96% of the LODE model cases were distinct (Constant Bite = 91%, Variable Bite = 89%, Bite Measurement Error = 96%) when considering all model parameters. Similarly, when we examined the number of confidence intervals that each parameter estimate overlapped with, state (θ) had less overlap than all Quadratic model parameters across all conditions (*p*’s < 0.001) while rate (*r*) had less overlap than the intercept and quadratic coefficients (*p*’s < 0.001; Table 1) across all conditions. Rate (*r*) had less overlap than the linear coefficient for the Bite Measurement Error condition (*p* = 0.026; Table 1) but did not differ from the linear coefficient for the other conditions. Overall, this suggests that the LODE model parameters were more distinct than the Quadratic model, which indicates it may have greater power to identify individual differences in parameter estimates.

#### 3.2.3. Goodness of Fit Index

There was little difference in parameter goodness of fit between simulations conditions (Table 1). While the intercept from the Quadratic model had worse fit for the Constant Bite than Bite Measurement Error condition (*p* = 0.020), the other conditions did not differ (*p*’s > 0.100). The conditions did not differ in fit for the other Quadratic parameters or the LODE model parameters (*p* > 0.126), suggesting that all are robust to sources of error. The linear coefficient of the Quadratic model had better fit than the doubling rate (*r*) from the LODE model (*p*’s < 0.003), however, rate (*r*) had better fit than the intercept and quadratic coefficients (*p*’s < 0.004). The linear and quadratic coefficients had better fit than state (θ) (*p*’s < 0.007), but state had a better fit than the intercept (*p*’s < 0.001). This indicates that the interpretable parameters (i.e., not the Quadratic intercept error term) show a mixed pattern when comparing goodness of fit between models. Overall, the interpretable parameters of both models had excellent goodness of fit.

#### 3.2.4. Cumulative intake curve error

While the LODE model always predicted feasible bite timings and sizes, the Quadratic model had between 1%–13% of simulated cases across simulation conditions with at least one non-feasible predicted bite value (see Supplementary Table 3). The Quadratic model also had two simulated cases fail to converge (>10% of predicted bites non-feasible): one each in the Constant Bite and the Bite Measurement Error conditions. These cases were excluded when calculating RMSE and pseudo-*R*^2^.

The amount of error in bite timing was very similar across simulation conditions for both RMSE (*p*’s > 0.090) and pseudo-*R*^2^ (*p*’s > 0.228; Table 1). For error in cumulative intake at each bite, RMSE did not differ between the Constant and Variable Bite conditions (*p*’s > 0.614), however, there was significant less error in the Bite Measurement Error condition than the Constant and Variable Bite conditions (*p*’s < 0.001; Table 1). The Quadratic model had less error in bite timing (*p*’s < 0.001) and a higher pseudo-*R*^2^ (*p*’s < 0.001) than the LODE model across all conditions. Overall, cumulative intake curve bite timings predicted using the LODE model had around one minute more error per bite compared to the Quadratic model (Table 1). While the Quadratic model had less error in cumulative intake at each bite than the LODE model for Constant and Variable Bite conditions (*p*’s < 0.001), the actual difference in RMSE was smaller than rounding error and, thus, do not appear different in Table 1. The models did not differ in intake RMSE for the Bite Measurement Error condition (*p* = 0.572) and there was no difference in intake pseudo-*R*^2^ across conditions (*p* > 0.499). All pseudo-*R*^2^ estimates were very high (minimum 0.88) indicating that recovered estimates for both models were able to predict almost all variability in the *true* cumulative intake and timing across bites. Together, this indicates that while the LODE model had relatively more error in predicting bite timing, both models show little error in their prediction of cumulative intake per bite and had excellent fit between the predicted and *true* cumulative intake curves for both cumulative intake and bite timing.

## 4. Discussion

This study provides evidence that cumulative intake curves can be characterized from video-coded data without the need for continuous weight measurement, which greatly expands their utility. This will alleviate the need for strict behavioral protocols that are common for continuous measurement of meal weight (e.g., not placing a utensil/hand on the plate), which can restrict the range of eating behaviors and may limit the ability to fully characterize individual differences or identify targets for intervention. For example, individuals with anorexia are more likely to tear or dissect foods and inappropriately use utensils (26, 27), and these behaviors would likely be restricted in protocols using continuously measured meal weight. Additionally, typical child eating behaviors (e.g., playing with food and using hands to eat) are often restricted. Expanding the characterization of cumulative intake curves to video-coded meals greatly expands the contexts and populations in which we can examine dynamic patterns of eating behavior.

To determine whether cumulative intake curves can be accurately characterized from video-coded bites, this study examined performance differences between data that simulated continuously measured meal weight versus video-coded bites. Both models had high goodness of fit and high predictive value for intake and bite timing. There was little difference in goodness of fit for bite timings between simulation conditions, however, there was significantly less error in the Bite Measurement Error condition than the Constant and Variable Bite conditions. Together, this indicates that measurement error incurred by using average bite sizes did not impair the ability to recover model parameters. While these results need to be validated in empirical studies, they indicate cumulative intake curves can be accurately characterized from video-coded meals.

While both the Quadratic and LODE models have been previously discussed and theoretically validated in the literature (4, 18, 19), this study was the first to formally validate parameter recovery. Both models showed excellent parameter recovery and goodness of fit. The higher-than-expected parameter recovery rates for 95% confidence intervals would result in a Type-I error rate slightly lower than would be expected (i.e., α < 0.05). That is, the confidence intervals provided a slightly conservative test of differences between estimates. Both models also had very high predictive value for both intake and bite timing, however, the Quadratic model showed relatively less error than the LODE model when predicting bite timing. While there were also significant differences in error for predicted cumulative intake between models, the size of the differences was within rounding error and too small to make a practical difference. These results validate the ability of both models to accurately estimate model parameters and cumulative intake curves.

While the models did not differ in their ability to recover parameter estimates, they did differ in the distinguishability of parameter estimates. The Quadratic model parameters were significantly less distinct than the LODE model parameters. Low distinctness or distinguishability suggests that some differences between true cumulative intake curves lead to very small changes in the estimated parameters. Greater differences in true cumulative intake curves (e.g., larger intervention effects or group differences) may be needed to derive statistically distinguishable parameter estimates from the Quadratic model than the LODE model. The extent to which this difference in distinctness impacts the power of point-estimates to detect individual differences in empirical studies still needs to be examined. The good distinguishability of LODE model parameters is promising and suggests the LODE model may be more sensitive to differences in eating behaviors between individuals or meals.

Similarly, associations between the Quadratic and LODE model parameters indicate that while the models capture shared information about cumulative intake curves, the LODE model may also capture unique information. Greater values for the initial state (θ) were associated with higher y-intercepts, more positive linear slopes, and more negative quadratic slopes from the Quadratic model. While both the initial state (θ) and the linear slope are thought to reflect initial rates of eating (18, 19), the state (θ) parameter was also associated with the quadratic coefficient, which is thought to reflect satiation (18, 19). In contrast, associations with the doubling rate (*r*) were smaller, suggesting that *r* may capture unique information that is not captured by θ or Quadratic model parameters. Future experimental studies are needed to understand the behavioral correlates of doubling rate (*r*) to better contextualize the dynamic information it captures.

The differences between the parameterizations of these two models may have useful implications for researchers. The independence of LODE model parameters allows them to capture unique information about the cumulative intake curve while the correlated Quadratic model parameters capture at least some amount of shared information. Additionally, while the LODE model can capture both the stimulation and satiating phases of eating, the Quadratic model can only capture one phase of eating at a time. While the cubic model performed well and would be able to capture two phases of eating (18), it would not resolve the issue of predicting non-feasible intake patterns (Figure 1). The Quadratic model had up to 13% of cases with at least one non-feasible value and two cases that failed to converge. In contrast, the LODE model did not predict any non-feasible values. We argue that although both models perform well, there may be some theoretical advantages to the LODE model.

This study provides initial evidence that cumulative intake curves can be characterized from video-coded meals and validates both the Quadratic and LODE models for characterizing cumulative intake curves. However, there are limitations that need to be address in future work. This study used simulated data; therefore, results need to be validated with behavioral data from adults and children. A limitation to the use of these models in empirical studies is the lack of easily accessible software. While the authors developed an initial suite of scripts that are freely available (21), a fully functioning package and tutorial are needed to improve accessibility. Lastly, additional simulation studies are needed to examine less conservative approaches to estimating confidence intervals.

Despite the remaining work to be done, this study advances the field of human ingestive behavior in three important ways: (1) it provides evidence that cumulative intake curves can be characterized from video-coded meals; (2) it validates the LODE model; and (3) it provides a formal comparison of the Quadratic and LODE models. Although the Quadratic and LODE models differ in their theoretical foundations, this study showed that both models accurately characterize cumulative intake curves. However, the LODE model had two advantages that may lead to improved ability to characterize individual differences: (1) it has independent parameters that capture unique information; and (2) recovered parameter estimates were more distinct, which suggests it is more sensitive to differences in cumulative intake curves. The ability to characterize of cumulative intake curves from video-coded meals greatly expands our ability to capture dynamic patterns of eating behavior in children and individuals with disordered eating. Additionally, eliminating the need for specialized hardware increases the ability of researchers to collect meal data quickly and cheaply using commodity cameras, or even by requesting participants record their own meals using their smartphone, greatly reducing the cost of data intake and potentially reducing participant burden. Although observational coding of meal videos is currently a time and resource intensive process after the data is collected, recent advances in automated coding of video will hopefully reduce this burden in the near future (28). The current work also extends the utility of these models to alternative approaches to bite detection such as the used of wearable devices (29). Together, this will improve our ability to identify patterns of eating behaviors associated with overconsumption and provide new opportunities for treatment.

## Data availability statement

The datasets presented in this study can be found in online repositories. The names of the repository/repositories and accession number(s) can be found below: https://github.com/alainapearce/LODEModel_SimStudy; https://osf.io/xfk5w/.

## Author contributions

AP and TB equally contributed to the design of the research, statistical analyses, wrote the manuscript, and had responsibility for the final content. AP conducted the simulations. Both authors contributed to the article and approved the submitted version.
