# Supplementary material for: Validation of computational models to characterize cumulative intake curves from video-coded meals
Source: Front Nutr. 2023 Jul 31;10:1088053. doi: 10.3389/fnut.2023.1088053 (PMC10425552; doi:10.3389/fnut.2023.1088053)
Supplement: Supplementary file 1 [file Data_Sheet_1.docx]

Supplementary Material

# Methods

## The LODE Model.

Due to a typesetting error, we solved the initial value problem presented in Thomas et al., (2017).

$$\frac{dE}{dt}= \left[ rE\left( t \right)+ \theta\right]\left( 1- \frac{E\left( t \right)}{E_{max}} \right), E\left( 0 \right)=0$$

E(t) is the amount of food intake, measured in grams, as a function of time *t,* measured in minutes and each of the model parameters $E_{max}$, $\theta$ and *r* are constants. The parameter $E_{max}$ is the total intake amount during the eating episode, $\theta$ is a non-zero initial rate of eating, which we term the *initial state* or *state*, and *r,* which we term *doubling rate* or *rate,* reflects eating duration as $\frac{1}{r}$ approximates the time it takes to double food intake.

The derivative can be simplified to:

$$\frac{dE}{dt}= \frac{-r}{E_{max}}\left( E+ \frac{\theta}{r} \right)\left( E- E_{max} \right), E\left( 0 \right)=0$$

Use separation of variables and integrate to get:

$$\int\frac{dE}{\left( E+ \frac{\theta}{r} \right)\left( E-E_{max} \right)}= \int\frac{r}{E_{max}}dt$$

Which give the implicit solution:

$$\frac{1}{E_{max}+ \frac{\theta}{r}} \left| n \right| \frac{E- E_{max}}{E+ \frac{\theta}{r}}= -\frac{r}{E_{max}}t+c$$

Solving for E = E(t) and applying the initial condition E(0) = 0, gives the explicit particular solution:

$$E\left( t \right)= \frac{E_{max} \left( e^{\frac{\left( E_{max} r+ \theta\right)t}{E_{max}}}-1 \right)}{e^{\frac{\left( E_{max} r+ \theta\right)t}{E_{max}}}+ \frac{E_{max} r}{\theta}}, E_{max}\neq-\frac{\theta}{r}$$

Multiplying the numerator and denominator by $\frac{1}{E_{max}}$ allows for further simplification:

$$E\left( t \right)= \frac{e^{\frac{\left( E_{max} r+ \theta\right)t}{E_{max}}}-1}{\frac{e^{\frac{\left( E_{max} r+ \theta\right)t}{E_{max}}}}{E_{max}}+ \frac{r}{\theta}}, E_{max}\neq-\frac{\theta}{r}$$

Setting $k=\frac{E_{max}r + \theta}{E_{max}}$ allows for further simplification and allows it to take a form similar to exponential decay:

$$E\left( t \right)= \frac{e^{kt}-1}{\frac{e^{kt}}{E_{max}}+ \frac{r}{\theta}}, E_{max}\neq-\frac{\theta}{r}$$

## Generating Model

### Child Microstructure Generation.

To establish reasonable sets of child microstructure behaviors, we used the faux package (1) to generate a multivariate normal distribution for number of bites, average bite size in grams, active eating time, and oral exposure per bite based on the central tendencies and variability reported in Fogel et al., (2017). To generate a dataset of microstructure behaviors, 500 sets of microstructure behaviors were sampled using mvrnorm (2). The following behaviors were then calculated using the sampled microstructure dataset: 1) total oral exposure, 2) total intake in grams, 3) eating rate in grams/minute, and 4) meal duration (see supplemental **Tables S1 and S2** for a comparison the sampled dataset and Fogel et al., 2017).

| Table S1. Mean and Standard Errors of Microstructure Behaviors for Fogel et al., (2017) and Simulated Data | | | | |
| --- | --- | --- | --- | --- |
|  | Slow, Mean (SE) | | Fast, Mean (SE) | |
|  | Fogel | Sim. | Fogel | Sim. |
| Number of Bites, N | 55.6 (2.50) | 48.95 (1.25) | 68.4 (2.50) | 58.28 (1.19) |
| Bite Size, g | 1.4 (0.10) | 1.59 (0.04) | 2.4 (0.10) | 2.83 (0.05) |
| Oral Exposure per Bite, sec | 20.1 (0.90) | 20.72 (0.38) | 15.6 (0.50) | 15.72 (0.33) |
| Active Mealtime, % | 75.0 (1.0) | 76.37 (0.56) | 76.0 (1.0) | 75.92 (0.54) |
| Total Oral Exposure, min | 15.1 (0.40) | 14.16 (0.25) | 15.2 (0.40) | 13.31 (0.26) |
| Fogel: data extracted from Fogel et al., (2017), g: grams; min: minute; sec: second; Sim: data from the simulation (see section 2.2.1 Generating Model). Sample Sized: Fogel et al., (2017) N = 386 children; simulated data N = 500. Median split: Fogel et al., (2017) = 6.41 g/min; simulated data = 7.47 g/min. | | | | |

The central tendencies and variation (**Table S1**) and correlation matrix (**Table S2**) for the 500 randomly selected cases of microstructure behaviors was similar to those reported in Fogel et al., (2017). However, the median of eating rate was slightly higher in the simulated data (Fogel et al., (2017): median = 6.41 g/min; Simulated Data: median = 7.47 g/min) and the mean number of bites was lower by about 10 bites in the simulated data for both the slow and fast eaters (Table S1).

| Table S2. Correlations Among Microstructure Behaviors for Fogel et al (2017) and Simulated Data | | | | | | | | | | | | | |  |
| --- | --- | --- | --- | --- | --- | --- | --- | --- | --- | --- | --- | --- | --- | --- |
|  | 1. | | 2. | | | 3. | | | 4. | | | 5. | | |
|  | Fogel | Sim. | Fogel | Sim. | Fogel | | Sim. | Fogel | | Sim. | Fogel | | Sim. |  |
| 1. Number of Bites, N |  |  |  |  |  | |  |  | |  |  | |  |  |
| 2. Eating Rate, g/min | 0.15 | 0.22 |  |  |  | |  |  | |  |  | |  |  |
| 3. Total Oral Exposure, min | 0.54 | 0.44 | -0.05 | -0.14 |  | |  |  | |  |  | |  |  |
| 4. Active Mealtime, % | 0.11 | 0.18 | -0.02 | 0.07 | 0.33 | | 0.32 |  | |  |  | |  |  |
| 5. Oral Exposure per Bite, sec | -0.58 | -0.66 | -0.25 | -0.4 | 0.02 | | 0.2 | 0.16 | | 0.07 |  | |  |  |
| 6. Bite Size, g | -0.42 | -0.33 | 0.55 | 0.52 | -0.01 | | 0.14 | 0.17 | | 0.15 | 0.54 | | 0.46 |  |
| Fogel: data extracted from Fogel et al., (2017), g: grams; min: minute; sec: second; Sim: data from the simulation (see section 2.2.1 Generating Model). Sample Sized: Fogel et al., (2017) N = 386 children; simulated data N = 500. | | | | | | | | | | | | | |  |

### Iterative Parameter Fit.

Parameter estimates were fit for both the Quadratic and LODE models using optim to minimize the -2 loglikelihood for intake. In order to ensure a stable parameter fit, an interative processes was used. The initial parameters for optim were the same for all simulations and were set to feasible parameters for both the Quadratic (intercept = 10, linear = 10, and quadratic = -1) and LODE models ($\theta$ = 10, *r* = 0.1). The iterative process consisted of the following steps:

1) The initial parameter fit was completed with optim and the initial parameters.

2) optim was re-run using the recovered parameters as the new initial parameters.

3) The recovered parameters from step 2 were compared to the initial parameters used in step 2 (i.e., the recovered parameters from previous fit).

4) If the newly recovered parameters in step 2 differed from the initial parameters used in step 2 (i.e., the recovered parameters from previous fit), steps 2-3 are repeated until a stable parameter fit is achieved.

## Parameter Recovery

### Parameter Confidence Interval Fit.

Data-driven likelihood profile confidence intervals (3) were fit by identifying the upper and lower bounds of the interval for each parameter using an optim in an iterative process until each boundary fit was stable. The upper and lower confidence bounds were fit separately using the likelihood ratio test to identify the parameter confidence bounds. The same iterative fitting process described in supplemental material section 1.2.2 Iterative Parameter Fit was used to achieve stable upper and lower confidence bounds.

### Parameter Feasibility.

The Quadratic parameters were then assessed to ensure they would predict feasible intake values by ensuring the minimum intake at time = 0 was less than total intake. Additionally, in the case of an inverted U-shaped curve (i.e., negative quadratic coefficient, *a^2^*), the model was considered feasible if 1) the predicted maximum intake was greater or equal to total intake and 2) both the predicted max intake and time at predicted max intake (i.e., the vertex) were positive. The LODE model parameters were also checked to ensure the derivative of the model (i.e., intake rate) could be computed. If either the Quadratic or the LODE model parameters were not feasible, the cumulative intake curve was re-generated with newly sampled bite timings until both models’ parameters were feasible.

### Non-Feasible Predicted Values.

As the recovered parameters may contain error, non-feasible predicted values were possible. While negative values were allowed to remain as error in predicted cumulative intake or timing, complex numbers were handled by computing the average of the adjacent *true* cumulative intake or bite timing values. If the complex number occurred for the first or last bite, the minimum or maximum of the *true* and predicted values was used, respectively.

# Methods

### Non-Feasible Predicted Cumulative Intake and Bite Timing (Table S3).

For the Quadratic Model, few cases had complex numbers as predicted timing and no cases had a complex number for predicted intake. However, between 10%-13% had negative predicted bite timing and 0%-3% had predicted negative cumulative intake values. Overall, two cases were non-convergent due to >10% of bites having negative predicted timing values.

| Table S3. Quadratic Model Non-Feasible Predicted Bite Cumulative Intake and Timing by Condition | | | | | | | | |
| --- | --- | --- | --- | --- | --- | --- | --- | --- |
|  | Timing | | | Intake | | | |  |
|  | Complex Number^a^ | Negative Value | | Complex Number^a^ | | Negative Value | |  |
| Cases with Non-Feasible Bites, % | | |  | |  | |  | |
| Constant Bite | 1% | 13% | | 0% | | 3% | |  |
| Variable Bite | 2% | 10% | | 0% | | 0% | |  |
| Measurement Error | 1% | 11% | | 0% | | 2% | |  |
| ^b^Bites per Case, Range | |  | |  | |  | |  |
| Constant Bite | 1-1 | 1-5 | | 0-0 | | 1-1 | |  |
| Variable Bite | 1-1 | 1-3 | | 0-0 | | 0-0 | |  |
| Measurement Error | 1-1 | 1-9 | | 0-0 | | 1-1 | |  |
| ^c^Fail to Converge, % | |  | |  | |  | |  |
| Constant Bite | 0% | 1% | | 0% | | 0% | |  |
| Variable Bite | 0% | 0% | | 0% | | 0% | |  |
| Measurement Error | 0% | 1% | | 0% | | 0% | |  |
| a: complex number indicates non-real number was computed (e.g., square-root of a negative value)  b: Bites per Cases where there is a non-feasible bite  c: a case failed to converge if >10% of bites had non-feasible predicted value | | | | | | | | |

### Distributions of Parameter Recovery Metrics

#### Parameter Estimate Overlap and Distinctness

Figure S1. Density distributions for parameter distinctness as measured by the number of confidence intervals (CIs) that recovered estimates overlapped with for the Quadratic and Logistic Ordinary Differential Equation (LODE) models. The green color reflects the Constant Bite Condition, the blue color reflects the Variable Bite Condition, and the red reflects the Measurement Error Condition.

#### Goodness of Fit

Figure S2. Density distributions of the goodness of fit of recovered parameters for the Quadratic and Logistic Ordinary Differential Equation (LODE) models. The green color reflects the Constant Bite Condition, the blue color reflects the Variable Bite Condition, and the red reflects the Measurement Error Condition.

#### Predicted Cumulative Intake Curve Error

Figure S3. Density distributions of intake and bite timing error from predicted cumulative intake curves. LODE: Logistic Ordinary Differential Equation; RMSE: root mean squared error. The green color reflects the Constant Bite Condition, the blue color reflects the Variable Bite Condition, and the red reflects the Measurement Error Condition.

# References

1. DeBruine L. faux: Simulation for Factorial Designs [Internet]. Zenodo; 2021. Available from: https://debruine.github.io/faux/

2. Venables WN, Ripley BD. Modern Applied Statistics with S [Internet]. Fourth. New York: Springer; 2002. Available from: http://www.stats.ox.ac.uk/pub/MASS4

3. Neale MC, Miller MB. The Use of Likelihood-Based Confidence Intervals in Genetic Models. Behav Genet. 1997;27(7):113–9.
